# Pulmonary Hypertension Workup Unraveling a Rare Anomaly in Turner Syndrome

**DOI:** 10.7759/cureus.112869

**Published:** 2026-07-17

**Authors:** Kit Yee Chu, Anthony Lutz

**Affiliations:** 1 Internal Medicine, Corewell Health Farmington Hills Hospital, Farmington Hills, USA; 2 Cardiology, Corewell Health Farmington Hills Hospital, Farmington Hills, USA

**Keywords:** congenital heart disease, multimodality cardiac imaging, partial anomalous pulmonary venous return, pulmonary hypertension, turner syndrome

## Abstract

Turner syndrome (TS) is associated with congenital heart disease, most commonly a bicuspid aortic valve and coarctation of the aorta; however, partial anomalous pulmonary venous return (PAPVR) remains an underrecognized cardiovascular anomaly. PAPVR, in which one or more pulmonary veins drain into the systemic venous circulation, can cause left-to-right shunting, right-sided chamber dilation, and pulmonary hypertension (PH). We report the case of a 51-year-old woman with TS and hypertension who presented with dyspnea and an estimated right ventricular systolic pressure of 55 mmHg on echocardiography. Right heart catheterization (RHC) showed severe PH, with a mean pulmonary artery pressure of 53 mmHg, a pulmonary capillary wedge pressure of 16 mmHg, and a pulmonary vascular resistance of 2.1 Wood units, along with unexpectedly high pulmonary artery oxygen saturations suggestive of a left-to-right shunt. Cardiac MRI demonstrated right-sided enlargement with a pulmonary-to-systemic flow ratio (Qp:Qs) of approximately 3.0 but no intracardiac defect. CT confirmed PAPVR, with the right superior and middle pulmonary veins draining into the superior vena cava and the left superior pulmonary vein draining into the brachiocephalic vein. She improved clinically with tadalafil and furosemide and was referred for surgical evaluation. In patients with TS and unexplained PH, an unexpectedly high pulmonary artery oxygen saturation on RHC should raise suspicion for an occult left-to-right shunt. When no intracardiac defect is identified, multimodality imaging with cardiac MRI and CT can reveal extracardiac shunt lesions such as PAPVR and guide timely referral for definitive management.

## Introduction

Turner syndrome (TS) is a chromosomal disorder characterized by complete or partial monosomy of the X chromosome. Patients with TS commonly exhibit a range of clinical features, including short stature, gonadal dysgenesis, and cardiovascular abnormalities [1]. The most well-reported congenital heart diseases in patients with TS are bicuspid aortic valve, coarctation of the aorta, and atrial septal defect [2]. However, other cardiovascular anomalies, including partial anomalous pulmonary venous return (PAPVR), are less frequently recognized in this population. PAPVR is a congenital defect in which one or more pulmonary veins drain into the systemic venous circulation rather than the left atrium, creating a left-to-right shunt [3]. This can result in chronic right-sided volume overload and progressively lead to pulmonary hypertension (PH) through increased pulmonary blood flow and vascular remodeling.

Earlier clinically based or echocardiographic cohorts reported PAPVR as an uncommon cardiac malformation in TS, accounting for approximately 2.9% of reported cardiac abnormalities [4]. In contrast, systematic cardiac MRI screening studies have identified a substantially higher imaging-detected prevalence of PAPVR. Gutmark-Little et al. identified PAPVR in 18% of adolescents and young adults with TS, most of whom had not been diagnosed by transthoracic echocardiography [5]. This distinction suggests that PAPVR may be underrecognized clinically, particularly when evaluation relies primarily on echocardiography.

Because PH often presents with nonspecific symptoms such as dyspnea and fatigue, underlying congenital shunts such as PAPVR may remain unrecognized until right-sided cardiac remodeling or hemodynamic abnormalities are detected. PH is defined hemodynamically by a mean pulmonary artery pressure (mPAP) >20 mmHg at rest, measured by right heart catheterization (RHC). Precapillary PH is further characterized by a pulmonary artery wedge pressure ≤15 mmHg and a pulmonary vascular resistance (PVR) >2 Wood units [6]. PH is classified into five WHO groups according to its underlying mechanism: pulmonary arterial hypertension associated with congenital heart disease (Group 1), PH due to left heart disease (Group 2), PH due to lung disease and/or hypoxia (Group 3), chronic thromboembolic PH (Group 4), and PH with unclear or multifactorial mechanisms (Group 5) [6]. Given its nonspecific symptoms and progressive nature, PH is frequently diagnosed at a late stage and is often associated with rapid clinical deterioration. Early recognition of PAPVR in patients with TS may help prevent or delay the development of PH.

In this report, we present a case of PH in a patient with TS who was subsequently found to have PAPVR, highlighting the diagnostic challenges, management strategies, and the importance of ongoing cardiovascular surveillance in this patient population.

## Case presentation

A 51-year-old woman with a history of TS and essential hypertension presented with progressive shortness of breath that began three years earlier after COVID-19 infection. Her dyspnea was initially intermittent but worsened over time, eventually occurring with mild exertion, such as eating or climbing stairs. She was initially evaluated by her primary care physician for shortness of breath and a cough productive of thick sputum. CT of the thorax revealed possible lingular pneumonia. However, imaging also demonstrated a right-sided aortic arch, markedly dilated pulmonary arteries, and cardiomegaly, prompting further evaluation with echocardiography and referral to cardiology.

The patient reported partial improvement after antibiotic treatment, but her shortness of breath persisted intermittently, particularly with exertion. She also reported occasional chest tightness and dizziness when bending over but denied leg swelling or significant chest pain. Echocardiography demonstrated severe right ventricular (RV) and right atrial dilation, with evidence of increased right atrial pressure suggested by bowing of the interatrial septum. The RV systolic pressure was estimated at 55 mmHg based on the tricuspid regurgitation jet velocity and estimated right atrial pressure, raising concern for significant PH. Color Doppler imaging did not demonstrate evidence of an intracardiac shunt.

Given the broad differential diagnosis for PH, further evaluation was pursued. A ventilation-perfusion scan showed no evidence of pulmonary embolism. Serologic evaluation was negative, including antinuclear antibody, anti-Scl-70 antibody, and HIV antigen/antibody testing, making autoimmune and infectious causes less likely. A sleep study was performed, revealing moderate obstructive sleep apnea, and auto-positive airway pressure therapy was initiated. Due to her history of intermittent chest tightness, coronary angiography was also performed and revealed nonobstructive coronary artery disease.

Given her persistent symptoms and unexplained right heart dilation, invasive hemodynamic evaluation was pursued. RHC revealed severe PH, with an mPAP of 53 mmHg, a pulmonary capillary wedge pressure (PCWP) of 16 mmHg, a left ventricular end-diastolic pressure (LVEDP) of 14 mmHg, and a PVR of 2.1 Wood units (Table 1).

**Table 1:** Measurements from the RHC. * CO was measured using the simplified Fick equation: $\mathrm{CO} = \frac{\mathrm{Estimated}\ \dot{V}\!O_2}{1.36 \times \mathrm{Hemoglobin} \times (SaO_2 - SvO_2)}$ ** In the absence of complete venous oximetry sampling, the derived CO and CI from the Fick equation and the calculated PVR should be interpreted with caution and are considered unreliable for definitive hemodynamic classification. CI, cardiac index; CO, cardiac output; LVEDP, left ventricular end-diastolic pressure; PA, pulmonary artery; PAP, pulmonary artery pressure; PCWP, pulmonary capillary wedge pressure; PVR, pulmonary vascular resistance; RA, right atrium; RAP, right atrial pressure; RHC, right heart catheterization; RVP, right ventricular pressure; SVR, systemic vascular resistance

Parameter	Measured value	Expected range	Units
RAP	14	2-8	mmHg
RVP	92/8	15-30/2-8	mmHg (systolic/diastolic)
PAP	92/33	15-30/4-12	mmHg (systolic/diastolic)
PCWP	16	6-15	mmHg
LVEDP	14	3-12	mmHg
CO*	18.6	4-8	L/min
CI**	11.27	2.5-4.0	L/min/m²
PVR**	2.1	<2	Wood units
SVR	404.3	800-1200	dyn·s·cm⁻⁵
Oxygen saturation - RA	83%	65-75	%
Oxygen saturation - PA	83%	65-75	%
Systemic arterial oxygen saturation	89%	95-100	%

Interestingly, the pulmonary artery oxygen saturation was unexpectedly elevated at 83%, whereas normal mixed venous oxygen saturation in the pulmonary artery is typically approximately 65-75%. A complete oximetry shunt run was not performed. Therefore, a discrete oxygen saturation step-up between chambers could not be assessed. However, the elevated pulmonary artery oxygen saturation, in the setting of unexplained right-sided chamber enlargement, prompted additional evaluation for a possible extracardiac left-to-right shunt. Complicating this picture, the patient also had mildly elevated LVEDP and PVR, suggesting a possible additional component of mild heart failure with preserved ejection fraction (HFpEF) contributing to the PH, potentially related to age and a history of primary hypertension. Liver ultrasound was unremarkable, making a hepatic source of shunting unlikely.

**Figure 1:**
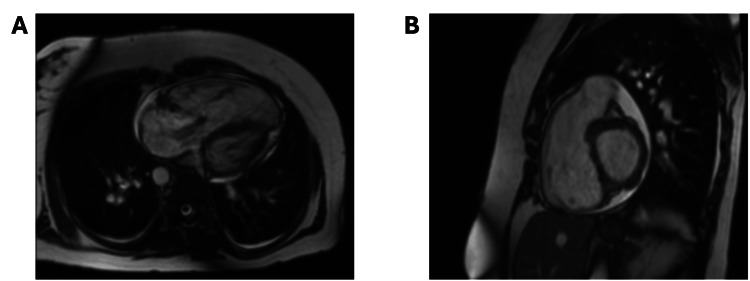
Cardiac MRI demonstrating severe RV dilation. (A) Four-chamber view showing marked enlargement of the RV relative to the LV. (B) Short-axis view demonstrating RV dilation with associated RV free-wall thickening, consistent with chronic pressure overload. LV, left ventricle/left ventricular; RV, right ventricle/right ventricular

A cardiac MRI was performed for further evaluation and revealed severe right-sided chamber enlargement with a pulmonary-to-systemic flow ratio (Qp:Qs) of approximately 3.0 by both flow and volumetric measurements, suggestive of a significant left-to-right shunt. The RV was severely dilated, with moderately reduced systolic function, an RV ejection fraction of 48%, and RV free-wall thickening of approximately 7 mm, consistent with chronic pressure overload (Figure 1).

**Figure 2:**
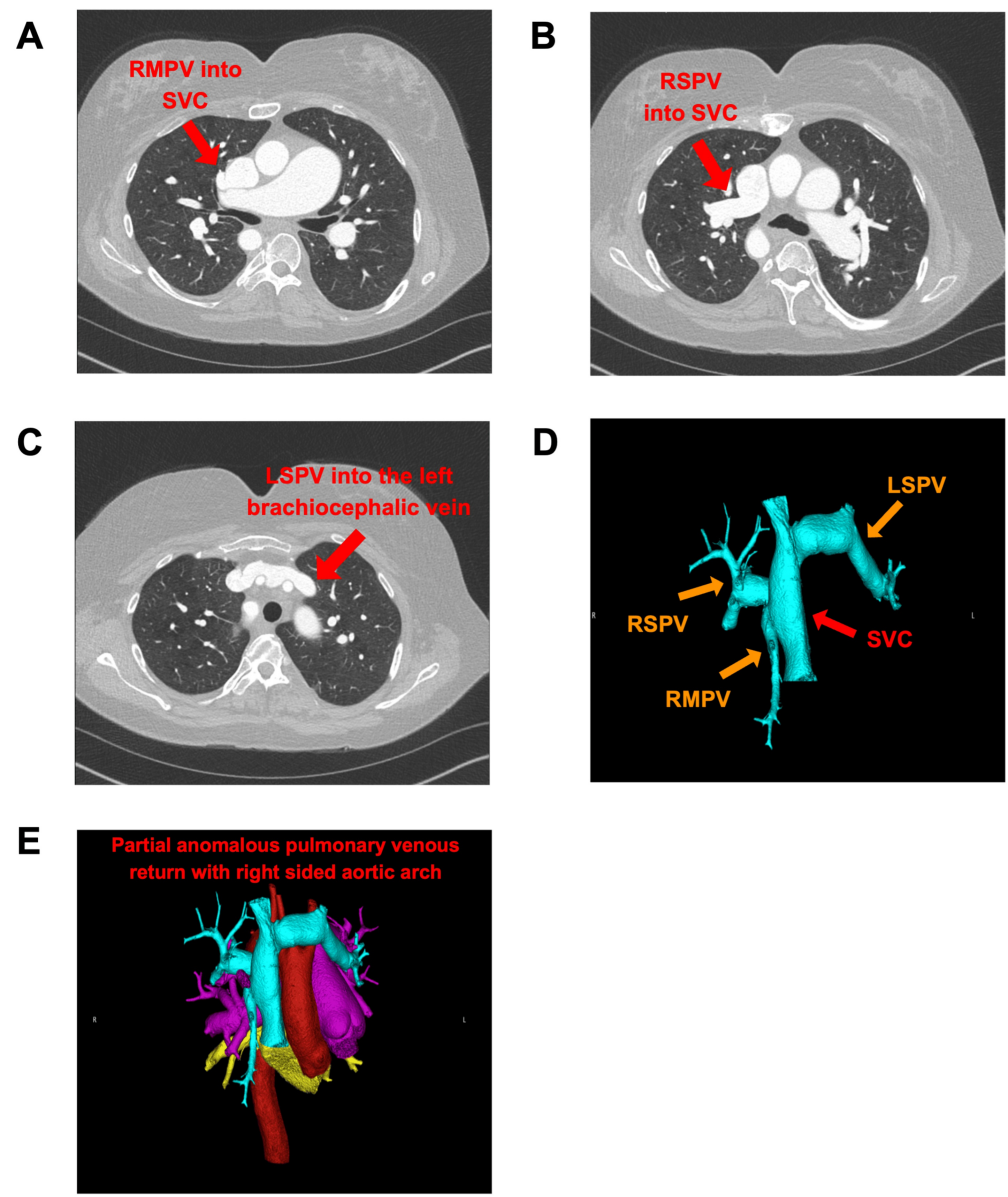
Chest CT and 3D reconstruction confirming PAPVR in TS. (A) Right middle pulmonary vein draining into the SVC. (B) Right superior pulmonary vein draining into the SVC. (C) Left superior pulmonary vein draining into the left brachiocephalic vein. (D) 3D reconstructed image demonstrating the connection of the pulmonary veins with the SVC (cyan). (E) 3D reconstructed image showing PAPVR with the SVC (cyan), aortic arch (red), and pulmonary veins (magenta). LSPV, left superior pulmonary vein; PAPVR, partial anomalous pulmonary venous return; RMPV, right middle pulmonary vein; RSPV, right superior pulmonary vein; SVC, superior vena cava; TS, Turner syndrome

In contrast, the left ventricle was normal in size and function (left ventricular ejection fraction 70%), with no evidence of myocardial fibrosis or edema, and all cardiac valves were structurally normal. In addition, no intracardiac shunt was identified on saturation band imaging. Notably, the right and left upper pulmonary veins were poorly visualized, raising a strong suspicion of PAPVR to the brachiocephalic veins, warranting further evaluation with CT angiography. She was then referred to an adult congenital heart disease specialist, who confirmed the diagnosis of PAPVR based on repeat echocardiography and chest CT imaging (Figure 2).

These studies demonstrated that the right superior and right middle pulmonary veins drained into the superior vena cava, while the left superior pulmonary vein drained into the left brachiocephalic vein. She was treated with furosemide and tadalafil 40 mg once daily, with notable improvement in dyspnea. Given the borderline elevated PCWP, tadalafil was used cautiously for symptomatic management of PH associated with PAPVR-related left-to-right shunt physiology during evaluation and optimization for definitive surgical repair. She responded well to pharmacotherapy, with improvement in dyspnea. She was subsequently referred to congenital cardiac surgery.

## Discussion

TS is associated with multiple cardiovascular abnormalities, including bicuspid aortic valve, coarctation of the aorta, aortic dilation, and congenital vascular anomalies. Although PAPVR has historically been considered uncommon in TS, earlier clinically based studies reported anomalous pulmonary venous connections in approximately 2.9% of cardiovascular malformations [4], whereas systematic cardiac MRI screening identified PAPVR in 18% of adolescents and young adults with TS [5]. This difference suggests that PAPVR may remain underrecognized when evaluation relies primarily on echocardiography.

PAPVR creates a left-to-right shunt that increases pulmonary blood flow and may lead to right-sided volume overload, pulmonary vascular remodeling, and PH [7]. In this case, echocardiography identified severe right-sided chamber enlargement but did not define the extracardiac anatomy. Cardiac MRI and CT angiography subsequently demonstrated the magnitude and anatomic source of the shunt, highlighting the importance of multimodality imaging in patients with TS and otherwise unexplained right-sided chamber enlargement or PH [3,8]. RHC provided an important physiologic clue. The initial pulmonary artery oxygen saturation was unexpectedly elevated at 83%, raising suspicion for a left-to-right shunt. However, because a complete oximetry shunt run was not performed, the initial study could not localize the source of shunting. A complete oximetry assessment with sequential sampling from the systemic veins and right-sided cardiac chambers can help identify and localize a left-to-right shunt [9].

A subsequent catheterization performed at an outside facility with more complete oximetry demonstrated elevated brachiocephalic vein, right atrial, and pulmonary artery oxygen saturations, with a pulmonary-to-systemic flow ratio (Qp:Qs) of 3.4:1 and a calculated left-to-right shunt of 6.9 L/min. These findings were concordant with the Qp:Qs of approximately 3.0 measured by cardiac MRI and with the PAPVR anatomy demonstrated by CT angiography. The large shunt also required careful interpretation of the Fick-derived measurements because pulmonary blood flow and systemic blood flow were not equivalent. In addition, the PCWP in this case was borderline elevated at 16 mmHg, indicating that the physiology was not consistent with isolated precapillary pulmonary arterial hypertension. Overall, the findings were most consistent with PH associated with congenital heart disease and PAPVR-related shunt physiology. Moderate obstructive sleep apnea, which had not yet been treated at the time of the initial RHC, was considered a possible additional WHO Group 3 contributor [6]. Lastly, HFpEF may also have contributed modestly to the PH because of the patient's age and history of hypertension.

Overall, previous reports have described PAPVR and complex pulmonary venous abnormalities in patients with TS [10]. Adult cases of PAPVR-associated PH have emphasized that delayed recognition may permit progression to advanced pulmonary vascular disease [11]. More recent reports have highlighted the value of multimodality imaging and invasive hemodynamic assessment in defining PAPVR-related shunt physiology and RV remodeling in patients with TS [8]. Collectively, these reports and the present case support consideration of PAPVR in patients with TS who have unexplained right-sided chamber enlargement, elevated right-sided oxygen saturations, or PH.

## Conclusions

This case emphasizes the importance of maintaining a high index of suspicion for congenital vascular anomalies such as PAPVR in patients with TS, which may lead to the development and progression of PH. Early detection is crucial, as management strategies differ depending on the underlying cause and may include surgical intervention in cases of significant shunting, underscoring the importance of raising awareness of PAPVR in this population.
